# Wales and Western Australia - Exploring Crisis Intervention for Children and Young People

**DOI:** 10.1192/bjo.2026.11633

**Published:** 2026-06-30

**Authors:** Ollie John, Alka Ahuja, Cayla Bellagarda

**Affiliations:** 1 Royal College of Psychiatrists Wales, Cardiff, United Kingdom; 2 University of Western Australia, Perth, Australia

## Abstract

**Aims:**

We aimed to compare findings from the first year of the NHS Wales 111 Press 2 mental health service and the CAMHS Crisis Connect (CCC) service that had been developed by the CAMHS Service in the Government of Western Australia.

We aimed to explore the commissioning and evaluation of these services, identify cross-national learning opportunities, and develop future models of mental health care in Wales that align with a “no wrong door” approach to support.

**Methods:**

We utilised a mixed-methods approach to evaluate service impact across two continents. For the Welsh 111 Press 2 service, researchers conducted a comprehensive review of 12 months of operational data (over 100,000 calls), utilising deprivation mapping against the Welsh Index of Multiple Deprivation (WIMD) to understand local need.

For the Western Australian CAMHS Crisis Connect (CCC) service, an Interrupted Time Series (ITS) analysis was employed to evaluate hospital resource utilisation - including emergency department (ED) presentations and inpatient admissions - between 2014 and 2024.

Findings were presented at a roundtable that was hosted by the Learned Society of Wales, and further visual illustration was provided to capture findings. The roundtable had input from experts by experience, policy professionals, researchers and clinicians from both Wales and Australia.

**Results:**

- NHS Wales 111 Press 2: Over 100,000 calls were received in the first year, with 99% of callers reporting a reduction in distress following triage. Approximately 50% of cases were resolved with self-care advice, while 10% required immediate crisis intervention.
- Western Australia CAMHS Crisis Connect: The ITS analysis demonstrated a 29% reduction in ED presentations for mental health concerns and a 28% decrease in inpatient admissions directly attributable to the service's introduction.
- Geographic Insights: Deprivation mapping in Wales revealed unexpected call patterns, such as high rates of suicidal ideation calls from affluent areas, highlighting the need for localised awareness strategies.

**Conclusion:**

We conclude that specialised mental health crisis lines can be effective in de-escalating distress and reducing the burden on acute hospital resources.

Future development should focus on co-production with young people, improving digital accessibility (e.g., via TikTok or WhatsApp), and utilising predictive analytics to manage demand during external events like climate hazards or economic shocks.

A formal partnership between Wales and Western Australia will continue to drive comparative research and data-driven service improvements.

